# Cesarean Scar Pregnancy and Successful Ultrasound-Guided Removal after Uterine Artery Ligation

**DOI:** 10.1155/2023/6026206

**Published:** 2023-04-21

**Authors:** Vito Leanza, Giosuè Giordano Incognito, Ferdinando Antonio Gulino, Attilio Tuscano, Monia Cimino, Marco Palumbo

**Affiliations:** ^1^Department of General Surgery and Medical Surgical Specialties, University of Catania, Catania, Italy; ^2^Department of Obstetrics and Gynaecology, Azienda di Rilievo Nazionale e di Alta Specializzazione Garibaldi Nesima, Catania, Italy

## Abstract

A correct management of cesarean scar pregnancy (CSP) is mandatory to avoid further complications. There is no consensus for the standard therapy and the most frequent methods used are not free from failures and sequelae. A 38-year-old woman was admitted referring amenorrhea lasting 9 weeks, pelvic pain, and vaginal bleeding. She had three previous cesarean sections. Transvaginal ultrasound showed a gestational sac of 16 mm in the cervico-isthmic site and inside the thickness of the uterine wall, and the dosage of beta-human chorionic gonadotropin was 12,770 mU/mL. A diagnosis of CSP was done, and an ultrasound-guided removal after uterine artery cervical branch ligation was performed. The follow-up was uneventful. Even if not yet codified in the literature, our therapeutic procedure should be considered in other similar cases in the future, as it potentially limits the possible iatrogenic problems and reduces intraoperative and postoperative bleeding to a minimum.

## 1. Introduction

Cesarean scar pregnancy (CSP) is one of the rarest forms of ectopic pregnancy, located in the scar of a previous cesarean section (CS) [1].

Early diagnosis is essential to avoid complications, including hemorrhage and uterine rupture. The first suspicion arises when a pregnant patient, who has had one or more CS previously [2], complains of symptoms, such as pelvic pain and vaginal bleeding. However, these clinical features are nonspecific and often are found in other obstetric conditions [3–5]. Furthermore, patients are asymptomatic in one-third of cases [6]. The differential diagnosis includes missed abortion, inevitable miscarriage, gestational trophoblastic disease, and cervical pregnancy [6]. The best confirmation is the visualization by transvaginal ultrasound (TVUS) of an empty uterine cavity with bright hyperechoic decidual stripes, an empty cervical canal, an intrauterine mass in the anterior part of the uterine isthmus, and the absence or thinning of the myometrium between the bladder and the gestational sac (GS) measuring less than 5 mm [6]. In addition to other functions [7], magnetic resonance imaging may be a further option for a more accurate diagnosis, but it was found to have a similar accuracy compared with TVUS and should be only reserved for inconclusive or difficult-to-diagnose cases [6].

Treatments vary from medical management to local ones and surgical approaches. There is no consensus for the standard therapy of this pathology and the most frequently used methods are not free from failures and sequelae [6].

## 2. Case Presentation

A 38-year-old woman was admitted to our emergency gynecologic unit referring amenorrhea lasting 9 weeks, inconstant pelvic pain, and vaginal bleeding that started 2 days earlier.

She had three previous deliveries, all performed by at-term CS: the first one in 2004 for a twin pregnancy, and the other two in 2009 and 2011. All CS occurred successfully and without any complications. Her family and personal histories were negative for any disease.

General physical examination was unremarkable and vital signs were within normal limits, with a heart rate of 90 bpm and a blood pressure of 105/60 mmHg.

The abdomen was treatable by superficial palpation and painful on the deep one at the hypogastric site. The uterus had a pasty consistency, and the cervix was closed; no adnexal tenderness or pelvic swelling during the bimanual visit was appreciable; the speculum examination did not show blood loss from the uterine cervix.

**Figure 1:**
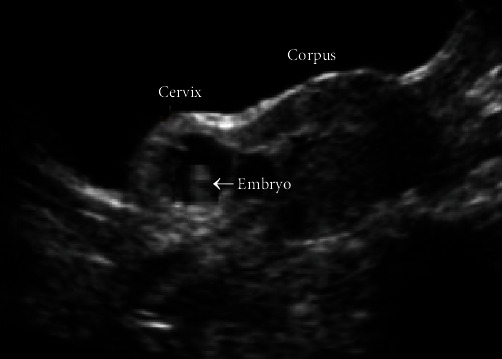
Ultrasound findings of scar pregnancy.

TVUS was carried out showing a uterus with a higher size (longitudinal diameter = 96 mm; latero-lateral diameter = 61 mm; and antero-posterior diameter = 48 mm). Uterine adnexa were regular in location and size with the presence of corpus luteum in the left ovary of 25 mm × 20 mm. No free fluid in Douglas' pouch was detected. In the cervico-isthmic site, inside the thickness of the uterine wall, a GS of 16 mm (corresponding to six gestational weeks) was detected with no embryonic echoes suggestive of heartbeat (Figure 1).

The dosage of beta-human chorionic gonadotropin (*β*-hCG) was 12,770 mU/mL, lower than the normal range for nine gestational weeks, ranging from 22,075 to 227,000 mU/mL. Considering the following findings: implantation on the site of previous cesarean wounds, stop of GS growth at six gestational weeks, absence of embryonic heartbeat on TVUS, and lower *β*-hCG values, a diagnosis of CSP was done.

**Figure 2:**
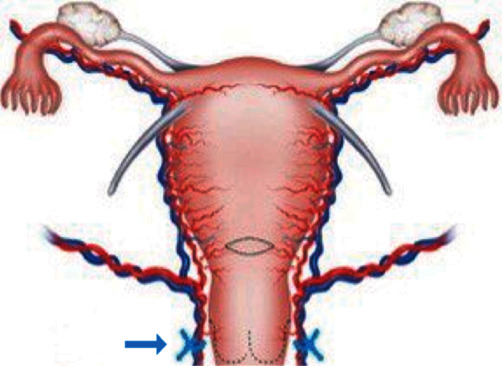
Bilateral ligature of the cervical branch of the uterine artery.

First, spinal anesthesia was carried out, and the patient was placed in a gynecological position with a sterile operating field. Cleaning and disinfection of the surgical site were done. By a transvaginal way, after visualizing the cervix and putting the tenacula, we started the procedure by performing bladder retraction, considering the multiple previous CS. A 2-cm horizontal incision was made in the anterior cervix about 1 cm beneath the estimated vaginocervical fold, and the bladder was retracted using a swab on a stick. The uterus was pulled towards the contralateral side of the intended ligature, to allow for ample working space and visualization of the vessel bundle. Thus, permanent bilateral ligature of the cervical branch of the uterine artery was performed using a synthetic absorbable thread (Vicryl, CT2, 2-0, Ethicon, Johnson & Johnson, Spreitenbach, Switzerland). An eight-distal loop-shaped stitch on each side of the late absorbable thread was applied, under the guidance of the index finger placed in the cervical canal and lower uterine segment, allowing the uterine artery pulsation. The whole procedure was then repeated with the contralateral vessel bundle (Figure 2).

The anterior vaginal wall and anterior cervical lip were reunited with a few interrupted stitches. A progressive dilation of the cervix up to 10 Hegar dilatators was carried out. A Foerster ring forceps was inserted into the cervico-isthmic cavity, and the entire GS was identified under ultrasound guidance and pulled out. Blood loss was negligible owing to the previous uterine ligation. Curettage of the uterine cavity ended the procedure. Finally, an utero-vaginal package was inserted.

The patient was kept under observation, and the postoperative course was uneventful. Slight blood loss was observed after removing the package the following day. On the third day, *β*-hCG was 223 mU/mL, and the patient was discharged following a TVUS control that showed a regular uterus, with normal endometrial thickness and cervix, absence of intrauterine blood clots or necrotic areas, and no free fluid in the Douglas pouch. The trophism of the cervix was found to be regular at follow-up.

A week after discharge, the blood test was normal and *β*-hCG values were 89 mU/mL. Four weeks later, *β*-hCG was unremarkable and menstruation resumed regularly. One year later, she delivered a healthy female newborn by elective and uneventful CS.

## 3. Discussion

The clinical case refers to a 38-year-old patient with an early CSP after a history of multiple previous CS.

The true incidence of CSP is unknown. It was estimated that values range widely from 1/800 to 1/2500 of pregnancies, with an increasing frequency related to the number of CS and advances in imaging [2].

Early diagnosis is essential to avoid the most fearful complications, such as uterine rupture and massive hemorrhage [6]. In the present case, after the admission of the woman to the emergency gynecologic unit, the suspicion of a pathologic course of pregnancy, based on the history of numerous previous CS and other suggestive symptoms including pain and bleeding, led to performing further investigation and a TVUS showed a GS inserted in a site of a very thin wall corresponding to cesarean incision.

At this point, it was necessary to choose the most suitable therapeutic option. Nevertheless, because of the limited number of reports on many cases, there is no consensus for the standard treatment of this pathology. Methotrexate (MTX) can be administered systemically or locally. However, it is a chemotherapy drug having a certain amount of toxicity, especially in the hepatic, hematopoietic, and immune systems, and some studies suggested that its administration carries a risk of heavy bleeding. Moreover, when MTX is not conclusive, the prosecution of pregnancy becomes risky owing to the further infiltration of the villi [8]. The presence of fibrous tissue surrounding the GS may limit the exposure of the trophoblast to systemic MTX and the need for additional treatment due to persistent fetal cardiac activity and/or increased *β*-hCG levels were reported [9]. Thus, we considered its use only as a secondary option, which could be taken into consideration in case of failure. Suction curettage combined with MTX was not associated with greater success rates in comparison with MTX treatment alone in the literature [10], and may cause bladder injury, especially when the distance between the myometrium surrounding the GS and the bladder is less than 3.5 mm [11]. Uterine artery embolization is an adjuvant treatment of CSP, and it can be used combined with local MTX or before curettage for bleeding prevention. However, it might be associated with decreased ovarian reserve, intrauterine growth restriction, premature delivery, and placental abruption, and requires an interventional radiologist, which may limit the availability of treatment [12]. Thus, compared with uterine artery embolization, the cervical branch of the uterine artery ligation may be simpler, less expensive, can be carried out vaginally, and requires no additional specialists.

A minimally invasive alternative for endogenic CSP treatment is transcervical resection by means of hysteroscopy [13]. In addition to other functions [14], hysteroscopy allows fast recovery, short follow-up, and a rapid decline of *β*-hCG to normal values [13]. However, hysteroscopic removal using a resector has a higher risk of bleeding, resulting in a more likely need to resort to hemostasis via electrocoagulation [13]. The uterine wall in correspondence of the cesarean scar is thinned and despite the operator's experience, the use of electrocoagulation could expose to the hazard of perforation of the uterus and adjacent organs, such as the bladder wall [15]. Laparoscopic removal may be performed for exogenic CSP [16]. In our case, this approach was excluded because the pregnancy was inside the isthmus and this method would have been dangerous for the risk of bladder lesions. Then, in agreement with the patient, it was decided to undertake a surgical procedure by choosing the technique considered more opportune for her clinical history, consisting of removing the entire GS using Foerster uterine forceps under transabdominal ultrasound guidance. The operation was successful. The ligation was not removed postoperatively, because it was performed using a synthetic absorbable thread and the collateral vasculature still allows for adequate blood supply to the uterus.

Despite the optimal postoperative condition of the patient, she was kept under observation for three days. Subsequent *β*-hCG measurements were taken up to 20 days later, at which time the values returned to nonpregnancy ones.

This case gives the opportunity to evaluate various aspects and advantages regarding the management chosen. It was characterized by rapid diagnosis and treatment, limiting to a minimum complication of the disease itself, and avoiding major surgery and possible iatrogenic problems due to possible overtreatment. The vaginally ligation of the descending uterine arteries, other than reducing the risk of bleeding during surgery, prevents postoperative bleeding. The procedure used is simple and surgery time-reducing, offering a short hospitalization. The transabdominal ultrasound check allowed a more rational and easy procedure, maintaining an excellent visualization of the cavity. Moreover, in the event of a failure of this procedure, it is not excluded to use other adjuvant medical or surgical techniques. Literature and clinical experience prove that uterine artery ligation is not associated with tissue devascularization due to the rich and complex blood supply of the womb. Therefore, we believe that this option, even if not yet codified, is very reasonable without complication. This should be considered in the event of other similar cases in the future.

## Data Availability

Data supporting this research article are available from the corresponding author or first author on reasonable request.
